# Making Moco: A Personal History

**DOI:** 10.3390/molecules28217296

**Published:** 2023-10-27

**Authors:** Sharon J. Nieter Burgmayer

**Affiliations:** Department of Chemistry, Bryn Mawr College, Bryn Mawr, PA 19010, USA; sburgmay@brynmawr.edu

**Keywords:** molybdenum enzymes, pyranopterin, molybdopterin, dithiolene, molybdenum cofactor, Moco

## Abstract

This contribution describes the path of my nearly forty-year quest to understand the special ligand coordinated to molybdenum and tungsten ions in their respective enzymes. Through this quest, I aimed to discover why nature did not simply use a methyl group on the dithiolene that chelates Mo and W but instead chose a complicated pyranopterin. My journey sought answers through the synthesis of model Mo compounds that allowed systematic investigations of the interactions between molybdenum and pterin and molybdenum and pterin-dithiolene and revealed special features of the pyranopterin dithiolene chelate bound to molybdenum.

## 1. Meeting Moco

It happened in 1980 while I was a graduate student at the University of North Carolina at Chapel Hill (UNC-CH) and doing research in organometallic chemistry with Joe Templeton: I was seduced by molybdenum. Templeton’s research revolved around organometallic reactions of Mo and W producing transformations of carbonyl and alkyne ligands, where these complexes incorporated dithiocarbamate chelates to fill out the metal coordination sphere [1,2,3,4]. During my search for information on Mo-dithiocarbamate chemistry, I encountered Ed Stiefel’s 1977 comprehensive review of Mo coordination chemistry in the *Progress in Inorganic Chemistry* series [5]—a chapter that I would later refer to as ‘the molybdenum bible’. It was from Ed’s review that I first learned about molybdenum in certain enzymes (how cool!) and where I met the molybdenum cofactor, Moco. The molybdenum cofactor, or Moco, refers to the catalytic active site in molybdenum enzymes. Moco circa late 1970’s was still minimally defined as follows [6]. It was known that it used Mo(4+), Mo(5+), and Mo(6+) oxidation states. Evidence from EPR was strong for thiolate ligands on the Mo atom, but the source of those S-donors was unknown. With the powerful new technique of extended X-ray absorption fine structure (EXAFS), it had been established that Moco had a variable number of Mo-oxo ligands that would soon be described as fitting a pattern of oxygen atom transfer reactivity [7]. Today we have much more information about the structure of Moco and how it varies among the three families of Mo-enzymes, about its biosynthesis, and the astounding breadth of substrates observed in nearly 100 molybdoenzymes across all biological kingdoms. The full history of the molybdenum cofactor was presented in the previous “State-of-the-Art in Molybdenum Cofactor Research” series by Professor Ralf Mendel in his review “The History of the Molybdenum Cofactor—A Personal View” [8]. Other contributions within this series provide more detailed information on different molybdenum and tungsten enzymes. In this personal history, I chart my path from meeting Moco through the twists and turns of my investigations to understand the chemical behavior of its unusual structure.

Fate or chance guided me to take a postdoctoral position in 1984 with Ed Stiefel, who was then at Exxon Research and Engineering. Arriving at the Exxon Corporate Labs, an imposing brick building positioned high on a hill in the farmland around Annandale, New Jersey, I felt like Dorothy was going to see the Wizard of Oz. While still a PhD student at UNC-CH, I had attended several of Ed’s energetic talks at ACS meetings, and each time I came away enthusiastic about the wonders of molybdenum and sulfur and in awe of Ed’s ability to make parallels between seemingly disparate areas such as molybdoenzymes in bacteria and molybdenum hydrodesulfurization catalysts in petroleum refining. My postdoc with Ed was the single best decision of my career, and it allowed me to make important connections and friendships at Exxon Corporate Research Labs.

## 2. Tormented by Pterins

**Figure 1:**
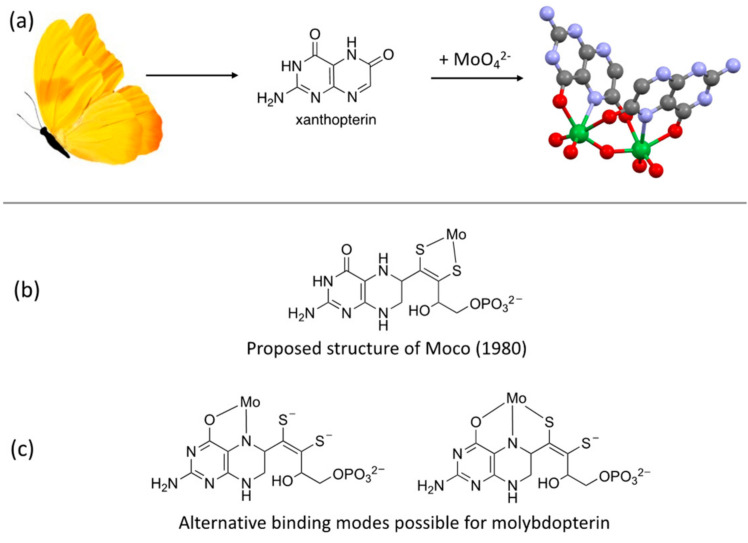
(**a**) The first molybdenum-pterin complex was prepared from the yellow butterfly wing pigment xanthopterin, producing a dimeric compound resembling a butterfly. (**b**) The proposed structure of Moco by Rajagopalan. (**c**) The demonstration that Mo could bind at the O and N chelation sites in a pterin suggested that dithiolene chelation might not be the only binding mode.

Once installed at Exxon in August 1984, Ed and I began to chart a plan. A pterin component of Moco had been recently discovered by Rajagopalan and coworkers at Duke University. Their recent experiments led to the proposal of a pterin-substituted dithiolene ligand in Moco that they named molybdopterin (Figure 1b) [9]. That the unknown sulfur donor ligand in Moco was a dithiolene chelate was a surprise—this would be the first example of a dithiolene ligand used in biology—but it was not an unprecedented structural motif in Mo chemistry. For Ed, the proposal of a dithiolene chelate in Moco made a lot of sense, as this was a familiar ligand he had extensively studied in *tris*-dithiolene complexes during his PhD research with Harry Gray [10,11]. The dithiolene presence in Moco was also a vindication for Ed, who had received early in his academic career a scathing review of a dithiolene-based research proposal wherein the reviewer announced something to the effect of “there was no such thing as a dithiolene in biology and there never would be”.

However, nothing was known concerning the chemistry between molybdenum and pterin. It was obvious to Ed and I that this was the direction I should take: investigate the chemistry between Mo and pterins in addition to finding a way to synthesize molecules closely related to the proposed molybdopterin structure. So I buried myself in the Exxon library to learn about the chemistry of pterins and pteridines. (After several weeks, Ed found me still reading pterin articles and told me I had to get into the lab!) This effort paid off with the preparation of the first example of molybdenum coordinated by a pterin ligand [12] using a bidentate O,N-donor binding site (Figure 1a), thereby demonstrating that Mo had options for binding in molybdopterin (Figure 1c) besides the proposed dithiolene chelate (Figure 1b) [9]. The dimeric Mo-pterin molecule seemed to be the punchline of a joke, since the pterin ligand used in the molecule, xanthopterin, is the yellow pigment of butterfly wings, and during the reaction with molybdate, it made a dimeric Mo complex that adopted a butterfly structure (Figure 1a). My investigations of pterins as ligands later expanded to other first-row metals [13] after learning that a metal-pterin complex was a cofactor of aromatic amino acid hydroxylases. At that time, both iron and copper had been identified in phenylalanine hydroxylases (though the copper version would later be refuted) [14,15].

However, my days in the Exxon labs were not all spent doing synthetic modeling work. I also had the opportunity to learn from biochemists how to isolate Moco and do *nit-1* assays, in addition to successfully accomplishing the NMF extraction of FeMoco from nitrogenase for NMR analysis, crystallization attempts, and other experiments [16]. My experiences in both areas yielded some humorous outcomes as the synthetic chemist encountered protein techniques. While doing nitrate reductase activity assays with Moco samples using the *nit-1* mutant nitrate reductase method, I was intrigued by the yellow species that formed when molybdate and dithiothreitol (DTT) were combined. Putting my synthetic chemist hat back on, I was able to synthesize and crystallize this material, and with the help of visiting scientist Avi Bino, determine the X-ray crystal structure to show that the yellow species was a dimeric [Mo_2_O_5_]^2+^ core bound to doubly deprotonated dithiothreitol [17].

In 1986, I accepted a position as assistant professor of chemistry at Bryn Mawr College outside of Philadelphia, and I initiated two new projects related to Moco model studies while continuing further exploration of my metal-pterin projects begun at Exxon. The first of these new projects sought to answer a question that had puzzled me since learning about the redox reactivity of pterins: in the proposed structure of Moco [9], how could a Mo atom in its highest oxidation state, Mo(6+), exist bound to a fully reduced tetrahydropterin? The second project sought to synthesize Mo compounds containing pterin-substituted dithiolene ligands through a coupling reaction between Mo(S_4_) groups and pterinyl alkynes. Both of these projects would become the basis of my research at Bryn Mawr over the next thirty-eight years.

## 3. Evidence for the Proposed Structure of Moco

**Figure 2:**
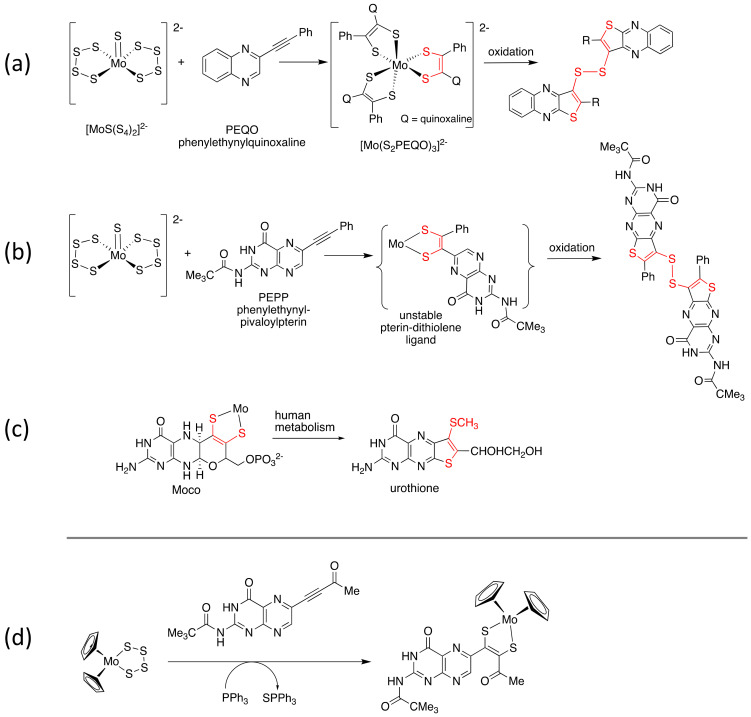
First synthetic approaches employed to prepare Mo(pterin-dithiolene) model complexes. (**a**,**b**) Use of [MoS_9_]^2−^ in reactions of quinoxaline and pterin alkynes to form dithiolene ligands. (**c**) Metabolic conversion of Moco to urothione. (**d**) Reaction of Cp_2_Mo(S_4_) and a pterin alkyne yields a pterin dithiolene ligand.

During my postdoc at Exxon, there were lots of discussions in Ed’s group about how to produce a model complex with a pterin dithiolene ligand coordinated to Mo. It was through such discussions regarding Moco modeling strategies with Bob Pilato, the post-doctoral researcher who followed me in Ed’s group, that we identified the approach of reacting a molybdenum tetrasulfide Mo(S_4_) group with an alkyne substituted by pterin to generate a pterin dithiolene ligand on Mo. Bob pursued this strategy in a collaboration between Ed Stiefel’s labs and those of Ted Taylor, the renowned pterin chemist at Princeton. From Bob’s organometallic background at Penn State, he chose to use Cp_2_MoS_4_ as the molybdenum tetrasulfide platform, while Taylor’s group developed the synthesis of a pterin alkyne closely related to Form A [18] that would be coupled to make the pterin dithiolene ligand (Figure 2d).

Simultaneously, I also began exploratory work at Bryn Mawr towards generating pterin-dithiolene ligands coordinated to Mo using the Coucouvanis methodology of reacting [MoS_9_]^2−^ with a pterin alkyne [19]. Prior to devoting effort to the arduous pterin synthesis, however, the initial studies of the Burgmayer group members, Ph.D. student Cheryl Soricelli and undergraduate Veronika Szalai, used an alkyne substituted by quinoxaline, a simple N-heterocyclic analog of pterin (Figure 2a).

The approach succeeded in coupling tetrasulfide ligands in [MoS_9_]^2−^ to a quinoxalylalkyne (PEQO) to produce the expected *tris*-dithiolene complex [Mo(S_2_PEQO)_3_]^2−^. The most significant result of this project, however, was not the successful dithiolene ligand formation but the identification of the oxidation byproduct produced from the synthesis (Figure 2a). It was eventually discovered that a number of oxidants would decompose the *tris*-dithiolene complex, [Mo(S_2_PEQO)_3_]^2−^, to the same mysterious yellow byproduct. While the *tris*-dithiolene complex failed to crystallize for X-ray analysis, the byproduct crystallized wonderfully, and we soon had a picture of its *bis*(quinoxalylthiophene) disulfide structure (Figure 2a). The proposed sequence of steps to the byproduct is dissociation of one S atom of the dithiolene chelate, rotation around the dithiolene C=C bond, attack of the uncoordinated S atom on the quinoxaline ring, oxidation of the incipient ring to a thiophene, and dissociation of the second S atom with oxidation to a disulfide. To our surprise, when we reacted the pterin alkyne analog PEPP, we did not obtain any of the expected *tris*-pterin dithiolene complex, [Mo(S_2_PEPP)_3_]^2−^, but instead isolated only the pterinylthiophene disulfide (Figure 2b). The formation of the pterinylthiophene byproduct indicated that the pterin dithiolene ligand was formed on Mo, but it dissociated and was oxidized by the above mechanism. We speculated that the pterin alkyne PEPP is perhaps too sterically demanding to permit three pterin dithiolenes to bind to Mo. The oxidative transformation of a Mo-dithiolene into a thiophene (Figure 2a,b) mirrored the known oxidation of Moco to Form B and its catabolic product urothione (Figure 2c), and thus this work was significant in corroborating Rajagopalan’s proposal of the dithiolene chelate in Moco [9]. This work was published in JACS in 1989 [20]. Meanwhile, Pilato, Stiefel, Taylor et al. beat me to the accomplishment of a synthetic pterin dithiolene Mo model complex, and in 1991 they published the preparation of Cp_2_Mo(pterin-dithiolene), the first Mo model complex including a pterin dithiolene ligand (Figure 2d) [21,22].

## 4. Discovering Non-Innocence and a Public Disagreement over Oxidation States

In parallel with pterin dithiolene syntheses, my early research career focused on exploring redox reactions between Mo(6+) and reduced pterins. It always seemed odd that Rajagopalan’s proposed Moco structure (Figure 1b) combined Mo in its highest oxidation state with a pterin in its most reduced, tetrahydropterin state. Would they not react to each other? My research team of Ph.D. student Heather Layton Kaufmann and a slew of undergraduates (Karen Kerr, Michelle Arkin, Laura Bostick, Kristen Everett, Kateri Paul, and Cory Rogge) found that a number of Mo(6+) reagents did in fact react with several reduced pterins. Our initial interpretation of these reactions was that a 2e^−^/2H^+^ redox reaction occurred to produce a Mo(4+) ion chelated by a semi-oxidized, quinonoid tautomer of dihydropterin [23]. We subsequently revised this initial interpretation after a lengthy study that taught me about the unusual covalency possible between metal and pterin and how to be suspicious of oxidation state assignments. This investigation covered the decade 1989–1999 and included a public disagreement with Berthold Fischer regarding the correct oxidation state assignment for Mo bound to reduced pterins. The next few paragraphs tell this story where, in the end, Bert and I were both wrong—or equally right.

**Figure 3:**
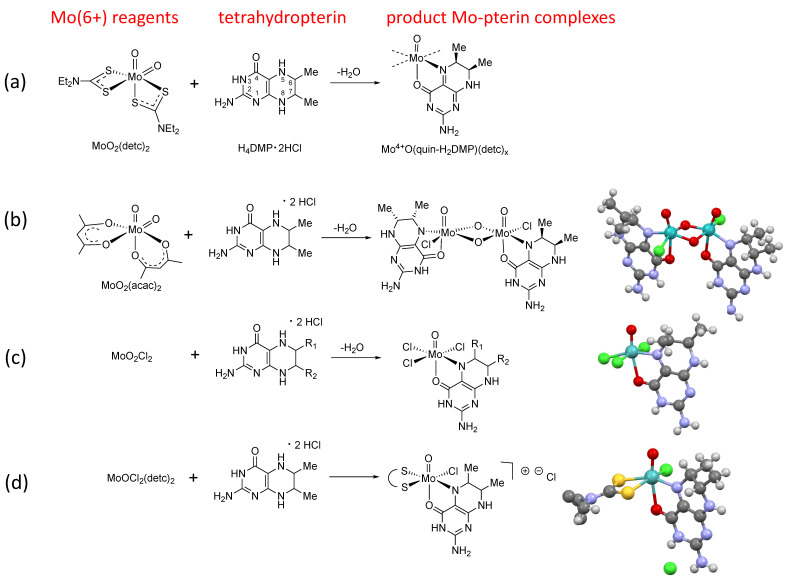
Reactions between various MoO_2_L reagents and reduced H_4_pterins yield mono-oxo Mo-pterin complexes, using (**a**) MoO_2_detc_2_, (**b**) MoO_2_acac_2_, (**c**) MoO_2_Cl_2_, and (**d**) MoOCl_2_detc_2_.

The Mo-reduced pterin oxidation state conundrum involved the reactions shown in Figure 3. Our initial study reacted MoO_2_detc_2_ with 6,7-dimethyl-5,6,7,8-tetrahydropterin (Figure 3a), which yielded a product exhibiting an intense absorption near 500 nm and a NMR spectrum suggesting the formation of a Mo(4+) ion coordinated by a dihydropterin in a quinonoid tautomeric structure [23]. When we replaced MoO_2_detc_2_ with MoO_2_acac_2_, we obtained the crystal structure of the dimeric Mo complex shown in Figure 3b, which was similarly assigned as a Mo(4+)-Mo(4+) dimer with each Mo ion coordinated by a quinonoid dihydropterin [24]. However, I was forced to reconsider those oxidation state assignments after Ed Stiefel admonished me to recognize that the Mo-Mo bond distance in the dimer had a very long Mo-Mo separation of 3.007 Å, which was inconsistent with a Mo(4+) dimer but indicated instead a Mo(6+)-Mo(6+) dimer. But did that mean that no pterin redox reaction had occurred and it was a tetrahydropterin bound to Mo(6+)?

We tried other dioxo-Mo(6+) reagents in reactions with several tetrahydropterins (Figure 3c,d), and in each case we observed the same intense absorption near 500 nm that we thought was characteristic of the Mo-reduced pterin structure [24,25,26]. We designed several reactivity studies to shed light on the oxidation state of the pterin in the dimer as well as on all the other products shown in Figure 3. The strategy was to displace the coordinated pterin by adding another chelating ligand and observe the dissociated pterin by NMR to determine its oxidation state [24]. The results of these reactivity studies were only consistent with an assignment of Mo(6+) bound by a deprotonated tetrahydropterin.

Turning to computational approaches for determining Mo and pterin oxidation state assignments, I employed two methods: the calculation of Mulliken charges using the extended Huckel molecular orbital (EHMO) method and the bond-valence-sum (BVS) method [27]. The outcome was that both methods predicted a formal molybdenum oxidation state midway between 5+ and 6+ [24,28].

**Figure 4:**
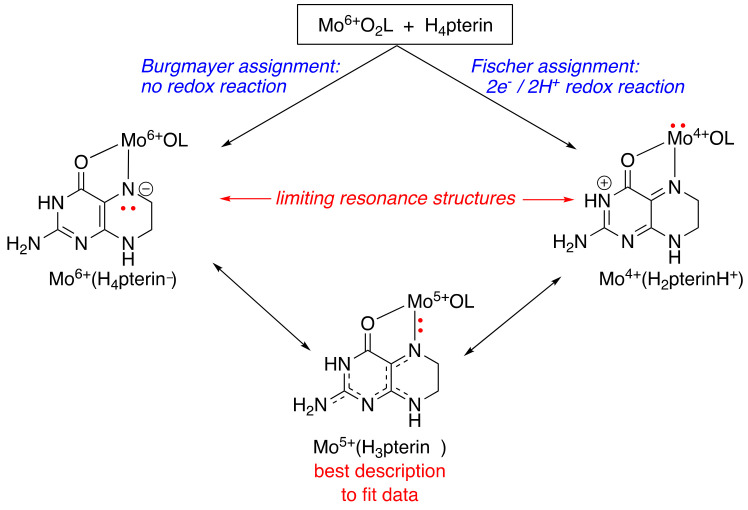
The reaction of dioxo-Mo(6+) complexes with tetrahydropterins was interpreted differently by the Burgmayer and Fischer groups. Further analysis by XPS and bond-valence-sum methods suggests the best description of the products is the intermediate assignment.

Meanwhile, Berthold Fischer in Bayreuth was likewise investigating the reaction of Mo(6+) reagents and tetrahydropterins; however, he assigned the products as Mo(4+) complexes of protonated quinonoid dihydropterin [29,30,31]. Following several public arguments we had concerning whose oxidation state assignment was correct, Bert proposed a collaboration involving X-ray photoelectron spectroscopy (XPS) to determine the charge on the Mo atom [32]. The outcome of this XPS collaboration still makes me laugh: we were both wrong, and the ‘true’ assignment was the compromise, right in the middle. These Mo-reduced pterin complexes were best described by both XPS (as well as my previous bond valence sum method) as Mo(5+) ions bound to a trihydropterin radical, where the diamagnetism of the complexes was explained by strong antiferromagnetic coupling of the Mo(5+) d^1^ electron and the pterin radical (Figure 4, bottom). We published all of this work and the collaborative results in three papers back-to-back in *Inorganic Chemistry* in 1991. For me, the key outcome from this work was recognizing the highly covalent nature of the Mo-pterin interaction and that the Mo-reduced pterin interaction was typical of other known ‘non-innocent’ ligands. Non-innocent ligands are characterized by a highly covalent interaction between a ligand and a metal, such that the redox state of metal and ligand is often ambiguous. A non-innocent ligand is also described as one that participates in partial electron transfer between metal and ligand. And such a description certainly fits the bill for the puzzling Mo-reduced pterin compounds.

Recognizing this covalent and non-innocent aspect of Mo-pterin interactions, my research group proceeded to investigate the outcome of doing the reaction the other way around; that is, we studied what happens when complexes of reduced Mo react with oxidized pterins [26]. As part of this new study, we reinvestigated a reaction between Mo(4+) and oxidized flavin first reported by Selbin [33] and subsequently rebutted by Sawyer [34]. In the end, we verified Selbin’s original interpretation by reproducing the synthesis and the spectroscopic data from his 1974 report, in addition to obtaining the X-ray crystal structure of MoOCl_3_(tmazH). Ultimately, this Mo(4+)-oxidized pteridine study showed a similar non-innocent behavior, where the products are best described as complexes of Mo(5+) bound to a protonated 1-electron reduced pterin radical.

## 5. The Pyranopterin Is Revealed

**Figure 5:**
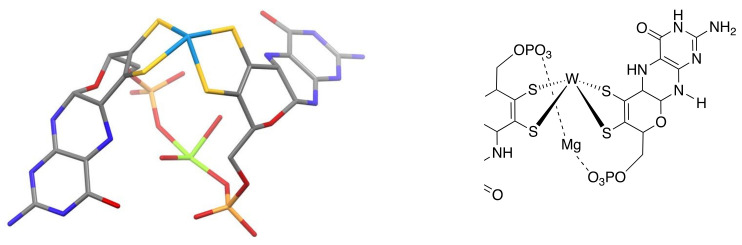
The first X-ray structure of the PDT ligand in Mo and W enzymes as observed in the *P. furiosus* W-AOR. protein.

The year 1995 was huge for molybdenum and tungsten enzymes, with the arrival of long-awaited protein structures revealing the active site structures [35,36]. Seeing the structure of the pterin-dithiolene ligand bound to W in *P. furiosus* W-AOR (aldehyde oxidoreductase) (Figure 5) [35] for the first time astounded me, then made me laugh. The pyranopterin structure was a surprise to everyone, but in fact Fate had already alerted me to its likelihood long before the crystal structures were published—it happened like this. I had been running a primitive Chem3D structure optimization program on Rajagopalan’s proposed Moco structure in order to include an image in an NSF proposal. Every optimization I did generated a cyclized pyran ring from the -CH(OH)CH_2_OPO_3_^2−^ sidechain. I would repeatedly break (computationally) the pyran ring open and restart the calculation, and it would reappear again and again. Sadly, I did not understand its meaning. If only I had been more knowledgeable about ring-chain tautomerization at this time, I could have gone on record to predict the presence of a pyranopterin in Moco before Rees et al. obtained the *P. furiosus* W-AOR protein structure.

### Is the Pyranopterin in Moco a Tetrahydropterin?

**Figure 6:**
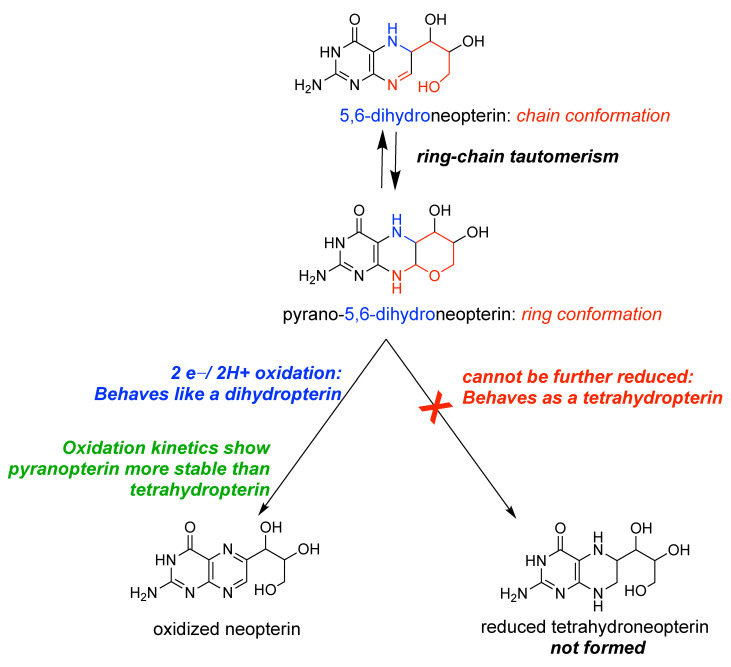
Summary of the unique redox behavior of a reduced pyranopterin.

Having spent years of effort to understand the outcomes of reactions between Mo(6+) and tetrahydropterins, the newly discovered pyranopterin structure in Moco threw me for a loop. Several of us synthetic chemists speculated on the ability of the pyran ring to open, which exposes a dihydropterin structure [37,38]. So what is the redox behavior of a pyranopterin—is it like a tetrahydropterin or a dihydropterin? In particular, I was intrigued whether the redox chemistry of a pyranopterin would fit observations Rajagopalan had made in the 1980’s when he and Gardlik investigated the redox state of the pterin in Moco [39,40,41]. Fortuitously, a simple pyranopterin had recently been reported by Viscontini [42] and Pfleiderer [43] as an unexpected product of a new synthetic pathway to neopterin. Pfleiderer and Soyka worked out that the pyranoneopterin was in equilibrium with both a ring-opened 5,6-dihydropterin and a furanopterin [43,44], thus setting the stage for such ring-opening chemistry in molybdopterin. Adopting this pyranoneopterin as a model for molybdopterin (Figure 6), graduate student Dori Pearsall and undergraduates Calies Sauk-Schubert, Shannon Blaney, and Eva Moore reproduced the synthesis and began reactivity studies with both oxidants and reductants. The conclusions drawn from these experiments, summarized in Figure 6, are that a pyranopterin exhibits chemistry distinct from simple pterins [45]. In short, the pyranoneopterin in Figure 6 behaves as a dihydropterin, where it can be oxidized by two electrons and lose two protons to form neopterin. However, we were unable to find a method to further reduce pyranoneopterin to 5,6,7,8-tetrahydroneopterin. Therefore, this pyranopterin behaves as if it were fully reduced.

## 6. The Dark Years Followed by a Move into the Light

Perseverence is perhaps the flip side of stubbornness, and both of these personality traits of mine explain the continued effort in the Burgmayer labs to accomplish the synthesis of a pterin-dithiolene model complex. Since the system of [MoS_9_]^2−^ and PEPP (Figure 2) failed to yield an isolable Mo-pterin-dithiolene complex, we next turned to the [MoOS_8_]^2−^ analog, which was shown by Coucouvanis to produce bis-dithiolene complexes [MoO(dithiolene)_2_]^2−^ from reactions with activated alkynes [46,47]. Applying this approach and using a pterin alkyne would then generate MoO-*bis*-pterin-dithiolene complexes to model Moco in the DMSOR family of enzymes. We had some success with this strategy and observed the formation of the desired [MoO(pterin-dithiolene)_2_]^2−^ species by ESI-MS analysis. However, this species was unfortunately unstable, decomposing under mild conditions instead to [MoO(dithiolene)(S_2_)]^2−^. Despite many attempts, we were unable to isolate any pure materials from these reactions to allow a systematic characterization of these *bis*-pterin dithiolene complexes. All our data were from material having a mixture of [MoO(pterin-dithiolene)_2_]^2−^, [MoO(dithiolene)(S_2_)]^2−^, and other polysulfides [MoO(dithiolene)(S_x_)]^2−^, thereby preventing publication of this work. In frustration, I called this period ‘the dark years’.

**Figure 7:**
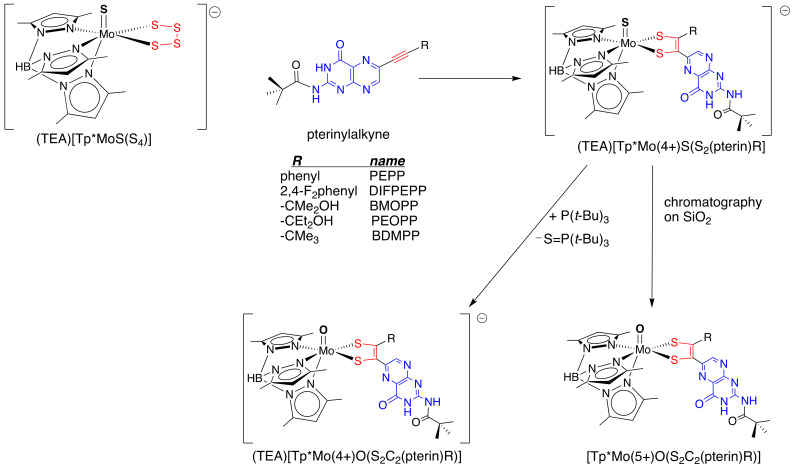
Use of the [Tp*MoS(S_4_)]^−^ route to pterin-dithiolene complexes.

Just as I had resigned myself to abandoning the synthetic approach of reacting Mo-tetrasulfide complexes with pterin-alkynes to produce Mo-pterin-dithiolene model compounds, I learned that Charlie Young’s group in Melbourne had reported that a monomeric tetrasulfide reagent [Tp*MoS(S_4_)]^−^ (Tp* is *tris*-(3,5-dimethylpyrazolyl)borohydride) [48] reacted with an activated alkyne to give a mono-dithiolene complex [Tp*MoS(dithiolene)]^−^ [49]. We immediately shifted gears to go into production of the [Tp*MoS(S_4_)]^−^ precursor and initiate studies of its reactions with various pterin-alkynes (Figure 7). Happily, this system worked great, and soon we had several examples of [Tp*Mo(5+)O(pterin-dithiolene)] complexes for detailed characterization [50]. The secret to success in this system is generating pure [Tp*MoS(S_4_)]^−^ without any of the oxo analog [Tp*MoO(S_4_)]^−^ contaminant, which does not react with the pterin alkynes. We spent over 20 years tweaking the [Tp*MoS(S_4_)]^−^ synthetic procedure to improve its efficiency, purity, and yield. Because we initially isolated the pterin dithiolene model complexes in the Mo(5+) state, we were unable to use NMR as part of a routine characterization. Fate intervened again by creating an encounter with Martin Kirk from the University of New Mexico at the 1998 Metals in Biology and a second meeting at the first Molybdenum and Tungsten Enzymes Gordon Research Conference in 2001. I asked Marty whether he would be interested in looking at these compounds using EPR and MCD [50]. He agreed, and this marked the beginning of our productive two-decade-long collaboration. In this, our first collaborative investigation, we were surprised to find that the presence of the pterin substituent on the dithiolene chelate had almost no effect on the EPR parameters as compared to those observed for the simpler benzene dithiolene bdt in [Tp*MoO(bdt)]. However, MCD spectroscopy revealed subtle differences between the pterin dithiolene complexes vs. [Tp*MoO(bdt)] that were interpreted to reflect an inherent energy difference between the S–C=C–S p systems of bdt and pterin dithiolene ligands.

**Figure 8:**
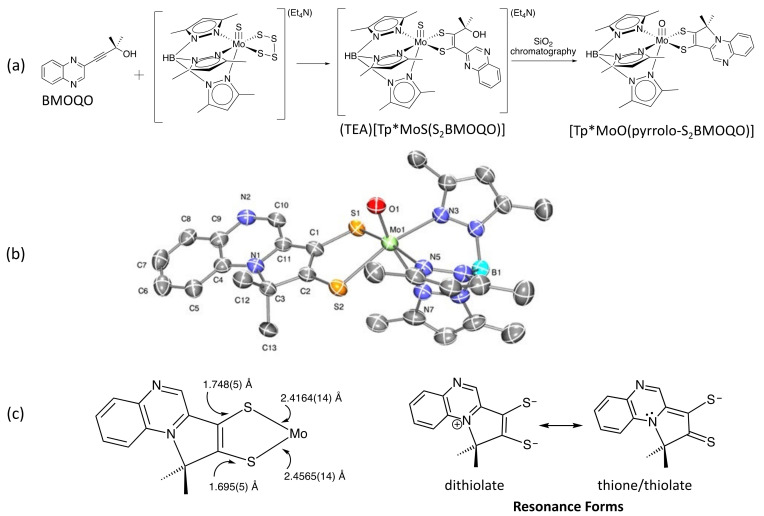
(**a**) Formation of a quinoxaline dithiolene complex [Tp*MoS(S_2_BMOQO)]^—^ and its conversion to a pyrrolo-quinoxalyldithiolene [Tp*MoO(pyrrolo-S_2_BMOQO)]. (**b**) X-ray crystal structure of [Tp*MoO(pyrrolo-S_2_BMOQO). (**c**) Asymmetrical Mo-S and C-S bond distances in dithiolene chelate that correspond to admixture of the two resonance forms at right.

While developing the pterin dithiolene model chemistry in Figure 7, Ph.D. student Kelly Matz also attempted the dithiolene-forming reaction using a quinoxaline group to replace pterin due to the easier synthesis. In the case of the quinoxaline dithiolene formed from BMOQO (Figure 8a), an oxidative dehydration reaction occurred during chromatographic purification to produce a novel *pyrrolo*-quinoxalyldithiolene Mo(4+) complex, which was unusually stable and crystallized quite nicely for us (Figure 8b) [51]. A significant feature of its structure is the asymmetry in the dithiolene chelate, where one C-S bond is markedly shorter than the other (Figure 8c). Marty Kirk did computational studies that were interpreted in combination with electronic and resonance Raman spectra data, which lead to a description of the pyrrolo-quinoxalyldithiolene chelate as having a substantial amount of a thione/thiolate resonance structure mixed with the dithiolate resonance structure that is expected for a dithiolene ligand [52]. The asymmetric resonance structure results from a redistribution of electron density from the dithiolene to the quinoxaline, leading to a dithiolene → quinoxaline intraligand charge transfer transition in the electronic absorption spectrum responsible for the intense blue color of the complex. This thione/thiolate description of the asymmetric dithiolene foreshadowed the important role of the thione/thiolate description of several pterin dithiolene ligands observed in later pyranopterin dithiolene model complexes [53,54,55].

## 7. The Three-Ring Circus of Pyranopterin

**Figure 9:**
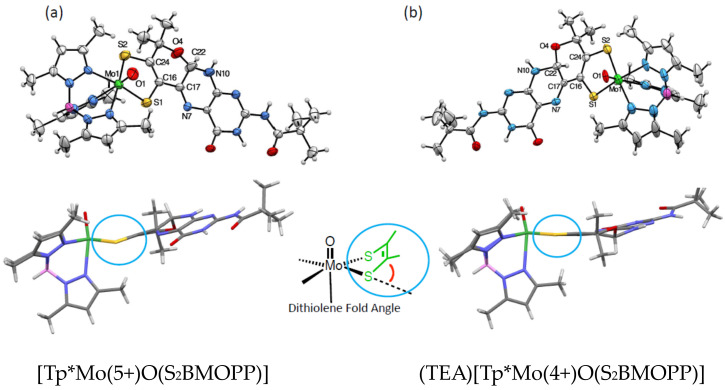
X-ray crystal structures of the first pyranopterin-dithiolene molybdenum complexes in both the (**a**) Mo(5+) and (b) Mo(4+) oxidation states. Below the ORTEP images are structures oriented to highlight the dithiolene fold angles (cyan circles) of (**a**) 26 deg for the Mo(5+) complex and (**b**) 5 deg for the Mo(4+) complex. The fold angle is determined using the planes defined by atoms Mo-S1-S2 and by dithiolene atoms S1-C-C-S2 dithiolene atoms.

The preparation of the pterin alkyne, nicknamed BMOPP (2-pivaloyl-6-(3-hydroxy-3-methylbutyne)pterin) (Figure 7 and Figure 9) was a game changer because its structure included a hydroxyl group in the alkyne sidechain that was positioned to allow cyclization, forming the pyran ring. To our great surprise and pleasure, the X-ray crystal structures (Figure 9) of the Mo(4+) and Mo(5+) complexes, (TEA)[Tp*Mo(4+)O(S_2_BMOPP)] and [Tp*Mo(5+)O(S_2_BMOPP)], showed that the pterin was present as a pyranopterin [53]! This result suggested that pyran cyclization in fact occurs spontaneously during pterin dithiolene synthesis without requiring any additional chemical intervention. The two structures exhibit the expected dithiolene fold angles of 5 degrees for the Mo(4+) complex and 26 degrees for the Mo(5+) complex.

**Figure 10:**
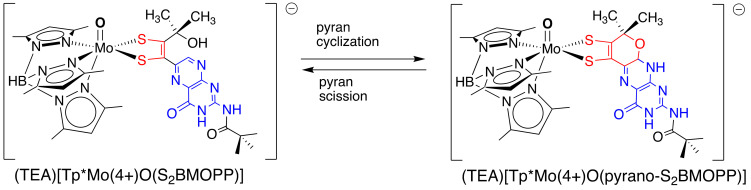
Reversible pyran cyclization and scission observed for TEA[Tp*MoO(S_2_BMOPP)].

Yet more surprises were in store for us from investigations of the ‘BMOPP’ model system. There were inconsistencies in the NMR spectra between [Tp*Mo(4+)O(S_2_BMOPP)]^−^ dissolved in acetonitrile vs. chloroform solvents that led us to realize that the complex could exist in both the pyranopterin, i.e., cyclized, and the non-pyranopterin, i.e., open, forms of the S_2_BMOPP pterin-dithiolene ligand (Figure 10). Ultimately, Ph.D. student Ben Williams showed that the solvent environment determined the most stable pterin conformation, where polar solvents favored the pyranopterin structure while non-polar solvents caused the open pterin form to predominate (Figure 10) [54]. Variable-temperature NMR studies yielded the thermodynamic parameters of the reversible cyclization, from which it was confirmed that the reversible pyran formation was a low-energy (∆G_act_ < 10 kJ/mol) process. Thus, we now have experimental evidence to confirm those speculations made several decades ago that pyranopterin might exhibit ring-opened forms [37,38]. This work was also relevant to certain structures of Moco observed in several protein structures in the dimethylsulfoxide reductase family (DMSOR), which is distinctive in possessing two PDT ligands coordinated to the Mo atom. An unusual form of Moco has been observed in just three of the many DMSOR proteins, where it is observed that one of the two pyranopterin dithiolene (PDT) ligands exhibits a pyran-opened conformation [56,57,58]. In the case of the *E. coli* NarGHI nitrate reductase that exhibits the ‘open’ PDT conformation, it had been proposed that the open form of PDT might be present to modulate the Mo redox potential, whereas the pyranopterin conformation positioned close to the electron transfer iron-sulfur cluster was proposed to be involved in moving electrons to the Mo ion after turnover [57].

## 8. The Electronically Unique Mo(4+)-Pyranopterin System

**Figure 11:**
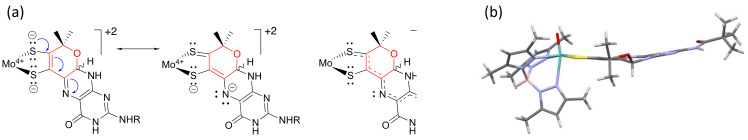
(**a**) Resonance structures of thione/thiolate (R = -C(O)CMe_3_). (**b**) View of co-planarity of dithiolene and pyranopterin groups in [Tp*Mo(4+)O(S_2_BMOPP)]^−^, where the torsion angle between S_2_C_2_ atoms of dithiolene and pterin plane is 9 deg.

The ‘BMOPP’ model system was the closest synthetic model to Moco in regards to the Mo-pyranopterin dithiolene (Mo-PDT) unit in Moco, and we proceeded to undertake a number of studies with Marty Kirk and his lab to explore its behavior. We first addressed the question of the electronic differences between an open pterin dithiolene and a pyranopterin coordinated to Mo: would the Mo ion feel the difference between these two pterin conformations? In the ‘BMOPP’ system, the open and cyclized forms are in equilibrium; therefore, to accomplish a study of electronic differences, we needed to synthesize a new pterin dithiolene that would only exist in the open conformation. Doug Gisewhite, a Ph.D. student in my lab at the time, took on this challenge and designed and synthesized the ‘BDMPP’ model bearing a *t*-butyl on the side chain (Figure 7) [59]. Comparison of the pyranopterin complex [Tp*Mo(4+)O(S_2_BMOPP)]^−^ to the open pterin complex [Tp*Mo(4+)O(S_2_BDMPP)]^−^ revealed a pivotal role of the pyran ring in switching on electronic communication between the Mo atom and pterin via the dithiolene chelate [59]. In our first report of the electronic difference made by a pyran ring in a Mo-pterin-dithiolene, we highlighted the +54 mV increase in the Mo(5+/4+) reduction potential in the pyranopterin complex and the strong charge transfer absorptions (ε > 10,000 M^−1^cm^−1^) between 300–500 nm present for the pyranopterin dithiolene complex, but not for the open-pterin dithiolene complex. Both of these differences were attributed to increased conjugation throughout the pyranopterin dithiolene ligand of [Tp*Mo(4+)O(S_2_BMOPP)]^−^ that is possible because the pyran ring enforces a near-planar conformation of the pterin plane and the dithiolene plane (Figure 9b). In effect, the pyran switch turns on electronic flow from the Mo-dithiolene unit to the electron-poor pterin. Computational analysis (DFT) from Marty Kirk’s group was invaluable in developing the picture (Figure 11) of how the coplanar pyranopterin and dithiolene groups lead to measurable outcomes for the Mo-pyranopterin dithiolene unit detectable by Fourier transform infrared spectroscopy (Mo=O bond strength), cyclic voltammetry (E(Mo(5+/4+)), and electronic absorption spectroscopy (intraligand charge transfer, ILCT). The electron flow from the dithiolene to the pterin is essentially a partial oxidation of the dithiolene chelate, where loss of electron density from a specific C-S bond is detectable as a shorter C-S distance in the X-ray structure of [Tp*MoO(S_2_BMOPP)]^−^ and is akin to the formation of a thione group, C=S (Figure 9a). The importance of the thione/thiolate nature of the pyrano-S_2_BMOPP ligand would become a recurring theme in further studies.

## 9. The Magenta Species

During more than 15 years of studying the ‘BMOPP’ pyranopterin dithiolene model system, we frequently saw intensely colored magenta species appear under different circumstances, and “the magenta species” label became somewhat of a joke in the group because it was used to define a fleeting and un-isolable material. However, graduate students Cassandra Gates and Haley Varnum managed to trap and study one of these magenta species, and it became the featured molecule in a recent paper [55] illustrating how pterin protonation makes use of the special pyranopterin-dithiolene conjugation to dramatically affect Mo behavior.

**Figure 12:**
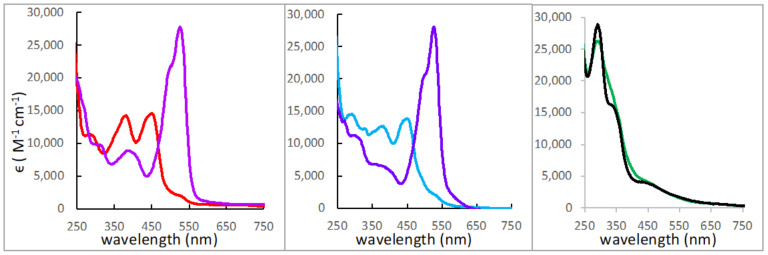
Room-temperature electronic absorption spectra of pyranopterin complexes [Tp*MoO(S_2_BMOPP)]^−^ and [Tp*MoO(S_2_PEOPP)]^−^ and the open pterin complex [Tp*MoO(S_2_BDMPP)]^−^ in ACN before and after addition of TFAA. Left: [Tp*MoO(S_2_BMOPP)]^−^ (red) and after adding 1 eq TFAA (purple). Center: [Tp*MoO(S_2_PEOPP)]^−^ (turquoise) and after adding 1 eq TFAA (blue). Right: [Tp*MoO(S_2_BDMPP)]^−^ (black) and after adding 1 eq TFAA (green).

It was discovered that the addition of acid (trifluoroacetic acid, TFAA) protonates the pyranopterin dithiolene complexes [Tp*MoO(S_2_BMOPP)]^−^ and [Tp*MoO(S_2_PEOPP)]^−^ (Figure 7), causing an immediate color change from yellow to magenta (Figure 12). In contrast, TFAA addition to a pterin-dithiolene complex without a pyran ring, such as [Tp*MoO(S_2_BDMPP)]^−^, resists protonation and exhibits almost no change in visible color and negligible change in its absorption spectrum (Figure 12). Our studies showed that pyranopterin protonation occurs at the pterin position N5 and enhances the electron-deficient character of the pterin, which in turn causes increased electron delocalization from dithiolene sulfurs to the pterin. This large change in the electronic structure of the dithiolene chelate and the Mo environment is revealed through the intense ILCT absorption at 526 nm (ε > 27,800 M^−1^cm^−1^) responsible for the magenta color. Computational analysis (DFT) led to the assignment of the ILCT absorption as dithiolene → pterin intraligand charge transfer (ILCT) transitions from one-electron promotion between dithiolene π HOMO-1 and HOMO-2 orbitals to a pterin π* LUMO [55].

We observed several surprising features of the protonated magenta species [Tp*MoO(S_2_HBMOPP)]. It exhibits much greater reactivity to air and other oxidants as compared to its parent complex [Tp*MoO(S_2_BMOPP)]^−^. A method was devised to isolate it as a solid, but unfortunately, it deteriorated over a few weeks even when stored in an inert atmosphere box. For this reason, all attempts to obtain an X-ray crystal structure were thwarted. We turned to the power of computation in collaboration with Marty Kirk and Jing Yang at the University of New Mexico, who used DFT to obtain an optimized structure of the magenta species, which revealed significant changes in the dithiolene. Notably, the torsion angle between the pterin plane (defined by the pyrimidine ring and N5) and the C_2_S_2_ atoms of the dithiolene chelate decreased by ~50% to 4 deg, indicating that pterin was essentially co-planar to the dithiolene. On inspection of the metrical parameters, we saw that the two dithiolene C-S bond distances were now even more different. Defining ∆(C-S) as the difference between thiolate (C-S) and thione (C=S) bond distances, ∆(C-S) increased to 0.066 Å, from which we inferred that the amount of thione/thiolate character in the dithiolene chelate was greatly increased. These incremental changes in Δ(C-S) inspired Marty to plot ∆(C-S) vs. % thione character for a number of related Mo(4+) dithiolene complexes. The plot revealed that the amount of thione/thiolate resonance in [Tp*Mo(IV)O(S_2_HBMOPP)]^−^ had increased by almost 40% as a consequence of pterin protonation. We concluded that the positive charge acquired at the protonated pterin N5 atom drove an electronic rearrangement that is a partial oxidation of the dithiolate chelate to a thione/thiolate chelate, significantly increasing the asymmetry within the dithiolene chelate. This interpretation in fact helped to explain yet another remarkable feature of the protonated pyranopterin complex: the Mo(5+/4+) reduction potential undergoes a whopping +315 mV positive shift as measured by cyclic voltammetry. Since a thione/thiolate chelate is expected to be a poorer electron donor to the Mo atom, it made sense that such a change in the dithiolene chelate would affect the Mo(5+/4+) reduction potential. The final intriguing feature of pyranopterin protonation in [Tp*MoO(S_2_HBMOPP)] is its facile redox reactions with oxidants in reactions that do not occur in the absence of pterin protonation. For example, [Tp*MoO(S_2_HBMOPP)] reacts immediately with the redox dye dichlorophenylindophenol (DCIP) and with air (O_2_), in contrast to [Tp*MoO(S_2_BMOPP)]^−^ which is unreactive to DCIP and is stable exposed to O_2_ for more than 5 h. It is presumed that the pterin acts as a proton shuttle for these proton-dependent reactions, thereby lowering the energy for the 2e^−^/2H^+^ redox processes. We also saw evidence for [Tp*MoO(S_2_HBMOPP)] reduction of DMSO to DMS, albeit in a sluggish reaction, in contrast to [Tp*MoO(S_2_BMOPP)]^−^ where no DMSO reduction was observed.

It is worth noting that the above chemical outcomes from pterin protonation are not observed in model Mo complexes where quinoxaline replaces pterin [60].

## 10. Ongoing Model Studies

We have not yet exhausted what we can learn from the Tp*Mo(pterin dithiolene) model system. Our current efforts are focused on two projects: (1) the reduction of the pyranopterin in model complex **1** and the accompanying study of the redox behavior of its reduced pyranopterin; and (2) the synthesis and characterization of a tungsten analog of model complex **1** [Tp*WO(S_2_BMOPP)].

**Figure 13:**
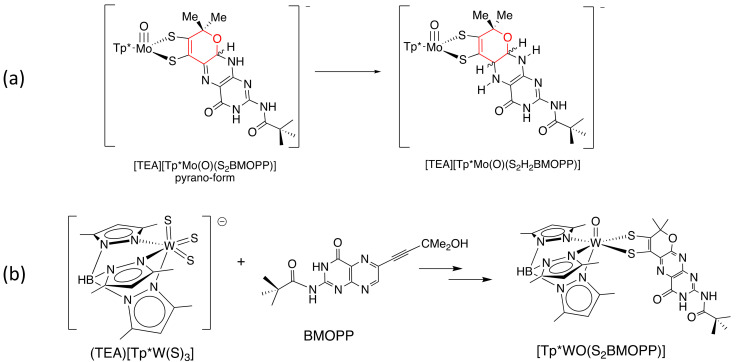
Current research projects underway in the Burgmayer labs. (**a**) Reduction of the pyranopterin to the dihydropyranopterin structure as found in protein crystal structures of Moco. (**b**) Synthesis of the first tungsten-pterin dithiolene complex.

We now have a methodology to successfully reduce the pyranopterin of model **1** to its fully reduced pyranopterin form (Figure 13a), thereby attaining a model complex with the same pyranopterin structure as observed in the protein X-ray structures of the majority of Mo enzymes. Our early results from redox titrations of this new reduced pyranopterin dithiolene complex already suggest this new model will provide additional surprises and insights, as evidence is accruing for both one electron and two electron redox pathways.

“*Can you do that with tungsten?*” That question is one frequently asked following our research presentations. We have recently addressed the issue of preparing a pterin dithiolene model for the tungsten cofactor, Tuco, by adapting the synthetic route for Tp*Mo-pterin dithiolene complexes with known tungsten analogs. Because tungsten chemistry differs somewhat from that of molybdenum, the pathway involves different intermediates where W is in oxidation states 6+ and 5+ rather than the Mo intermediates that all remain in the 4+ state (Figure 13b). The initial isolated W-pterin dithiolene products are 5+ species.

## 11. The Mo-Pyranopterin-Dithiolene Structure Is an Indivisible System

Two major points have emerged from our studies on the Tp*MoO(pyranopterin-dithiolene) model system. First, there is a high degree of electronic communication throughout the Mo-S_2_C_2_(pyranopterin) structure. This is indicated by the intraligand charge transfer transitions observed spectroscopically for every pyranopterin Mo complex. In contrast, such conjugation is largely absent in the open pterin model complexes, consistent with the absence of ILCT transitions in those complexes. The conjugation is an important feature of the Mo(pyranopterin-dithiolene) structure because it accesses long-range communication. For example, pyranopterin protonation at N5 four bonds away from the Mo atom causes >+300 mV change in the Mo(5+/4+) reduction potential.

The second major point is that the pyranopterin position adjacent to a dithiolene is special. Specifically, it is the pyran ring that imparts the co-planarity of the semi-reduced pyrazine ring of pyranopterin and the dithiolene chelate, and it is the semi-reduced pyrazine ring of pyranopterin that has the unique ability to turn on the conjugation between dithiolene and pterin that affects Mo behavior. As a result, only this pyranopterin state can access the thione/thiolate oxidized form of dithiolene, thereby making use of the multiple redox states of dithiolene. This special ability of pyranopterin is in striking contrast to that of reduced pyranopterin, which has a saturated pyrazine ring that is neither capable of pterin-dithiolene conjugation nor facile electronic communication with the metal ion.

Other subtleties emerge from our model studies. The reversible pyran ring cleavage that leads to an open pterin will necessarily cause the pterin to rotate out of plane with the dithiolene ligand to avoid a steric clash [59], thus eliminating the pterin-dithiolene conjugation and effectively switching off electronic communication from the pterin through the dithiolene to the Mo atom. Ring opening additionally changes the electronic structure of the pterin, making it oxidized rather than semi-reduced [55]. It also needs to be underscored that the electronic communication through the conjugated Mo(pyranopterin dithiolene) structure is specific for the Mo(4+) oxidation state. Negligible thione/thiolate character in the dithiolene is indicated for the Mo(5+) state since the C-S bond distances of the dithiolene differ by only 0.01 Å [52], hence little electron density transfer from dithiolene to pterin has occurred. This difference in the dithiolene character of Mo(5+) vs. Mo(4+) model compounds has an interesting connection to the dithiolene fold angle that correlates with the Mo oxidation state [61,62]. Only for the Mo(4+) state will the dithiolene fold angle be approximately 0 deg, making the redox active d(xy)orbital coplanar with the dithiolene plane. The Mo(4+) state is then poised for coupled electron proton transfer (CEPT) from the Mo ion through the pterin dithiolene ligand to nearby redox partners, triggered by proton transfer from a neighboring residue to the pyranopterin.

In writing this personal history and looking over the trajectory of my nearly four decades of investigations into Moco modeling, it becomes apparent how the earlier work on redox ambiguity and ligand non-innocence in Mo-pterin complexes set the stage for redox ambiguity in the Mo-pyranopterin dithiolene model compounds that we are just now beginning to appreciate and understand. All three components of Moco—the Mo ion, the dithiolene chelate, and the pyranopterin—form a unique system of electronic communication that functions to tune Moco reactivity within the enzyme.

## 12. Final Thoughts

I admit to feeling a certain amount of satisfaction at having accomplished what I set out to do four decades ago after my first encounter with the pterin component in Moco: synthesize pterin-containing models of Moco to determine its unique chemical behavior. The work that has been summarized in this personal history occurred at a small liberal arts college with predominantly undergraduate students. As I come to the close of my personal history, the overwhelming sentiment is gratitude. Gratitude for the hard, smart work of Ph.D. students and the more than 140 undergraduate students—too numerous to name—who have been the backbone of this ambitious synthetic effort. Gratitude also for being a part of such a collaborative, encouraging, warm, and welcoming community of scientists who have been similarly seduced by Mo (or W) enzymes. I have immensely enjoyed your company and the diversity of scientific approaches shared at MoTEC meetings over the past 20 years that have zigzagged from Brighton to Oxford, to New Hampshire, then Italy, to Alberta, Sintra, Balatonfüred, Santa Fe, Potsdam, and Indianapolis.

## Data Availability

No new data was created for this article.
